# The Effect of Three Daily Servings of Full-Fat Dairy for 12 Weeks on Body Weight, Body Composition, Energy Metabolism, Blood Lipids, and Dietary Intake of Adults with Overweight and Obesity

**DOI:** 10.1016/j.tjnut.2026.101373

**Published:** 2026-01-22

**Authors:** Gerald Harvey Anderson, Corrina Zi Chen Zhou, Shirley Vien, Marisa Soo, Shekoufeh Salamat, Maryam Akbarifakhrabadi, Larissa Chomka, Priya Kathirvel, Ferhan Siddiqi, Hrvoje Fabek, Bohdan Luhovyy

**Affiliations:** 1 Department of Nutritional Sciences, Temerty Faculty of Medicine, University of Toronto, Toronto, ON, Canada; 2 Department of Applied Human Nutrition, Mount Saint Vincent University, Halifax, NS, Canada; 3 Department of Medicine, Dalhousie University, Halifax, NS, Canada

**Keywords:** full-fat dairy, body weight, body composition, energy metabolism, dietary intake, Canada’s Food Guide, dietary counseling, dietary intervention, obesity

## Abstract

**Background:**

Habitual dairy consumption reduces risk factors for obesity and its associated characteristics of the metabolic syndrome.

**Objectives:**

This study aims to describe the effect of adding 3 daily servings of full-fat dairy to the diet of adults with overweight and obesity, counseled to follow Canada’s Food Guide (CFG).

**Methods:**

A 12-wk single-blinded, parallel, randomized study was conducted in 74 participants [age: 36.55 ± 1.04 y; body mass index (BMI): 29.34 ± 0.43 kg/m^2^] assigned to 1 of 3 groups: *1*) low dairy energy restriction (LD-ER): 500 kcal restriction with ≤1 serving of low-fat dairy, *2*) 3 dairy energy neutral (3D-EN): 500 kcal restriction replaced by 3 servings of full-fat dairy, and *3*) 3 dairy ad libitum (3D-AL): no energy restriction with 3 servings of full-fat dairy. Changes in physiological outcomes and dietary intakes were measured over 12 wk.

**Results:**

Body weight and BMI were reduced by treatment (*P* < 0.05) in LD-ER over the 12 wk (*P* > 0.05). In 3D-AL, a decrease (0.25 ± 0.34 cm) in hip circumference (*P* < 0.05) and in systolic blood pressure (2.72 ± 2.18; *P* < 0.05; SBP) was found at week 12. SBP also decreased in LD-ER (*P* < 0.05). Triglycerides increased in all groups at week 4 (*P* < 0.05) but returned to baseline by week 12. Neither treatment nor time affected waist circumference, fat and fat-free mass, resting metabolic rate, fasting blood cholesterol, and urine creatinine and urea (*P* > 0.05). Protein and calcium (*P* < 0.04) intakes were increased with time in 3D-EN and 3D-AL but not in LD-ER. Compliance with CFG, assessed by a food tracker, increased with time (77% by week 12).

**Conclusions:**

Frequent and daily consumption of full-fat dairy as part of a healthy diet is consistent with CFG.

This study was registered at clinicaltrials.gov as NCT04399460 on 22 May, 2020 (https://www.clinicaltrials.gov/study/NCT04399460).

## Introduction

Obesity and cardiometabolic disorders are prevalent within the Canadian population, with over 2.6 million adults diagnosed with cardiovascular disease and more than a third considered obese. Collectively, this poses an annual burden of $35 billion on the healthcare system [[1], [2], [3]]. These conditions are closely linked with weight and adiposity as excess body fat [4].

Body weight (BW) and composition are strongly influenced by diet [5,6]. Dairy is the second largest agricultural industry in Canada and provides a rich source of macronutrients and micronutrients [7,8]. It has been widely documented in many observational and randomized controlled trials, including low-fat and high-fat dairy, to be positively associated with lower BW, reduced waist circumference (WC), and more favorable blood lipid markers [[9], [10], [11], [12]]. However, there has been a decrease in dairy milk consumption among Canadian consumers from 70.2% in 2004 to 56.1% in 2015, with consumers choosing partly skimmed milk (1%–2%) over full-fat dairy (3.25%) [13,14]. A primary reason given for this shift is the association of animal-based foods with negative cardiometabolic outcomes, and the promotion of plant-based diets and alternatives [15]. As well, most nutrition recommendations advise against consuming full-fat dairy products, saturated fats, and food products from animal sources [9,16,17]. In contrast, a recent expert panel concluded that there is little evidence to support the differentiation between regular-fat and low-fat dairy foods in dietary guidelines for both adults and children [18].

The 2019 Canada’s Food Guide (CFG) moved away from its previous nutrient-based guidance with a goal to reduce intake of foods associated with chronic diseases. It encourages the consumption of plant-based foods and proteins and provides no quantitative recommendations for dairy intake [19]. In contrast, the Dietary Guidelines for Americans explicitly recommend 3 servings of low- or no-fat dairy a day for adults and specify that plant-based dairy alternatives (e.g., almond, rice, coconut, oat, and hemp) are not included in the dairy group, except for fortified soy products [20]. However, neither recognizes recent evidence that full-fat dairy may be beneficial. Full-fat dairy was inversely associated with central obesity compared with low-fat dairy in a 12-y follow-up study within a male cohort [11]. Another 12-wk study in individuals with metabolic syndrome found that 3-daily servings of either low-fat or high-fat dairy did not increase fasting serum cholesterol and triglycerides (TG) compared with a low dairy diet [12]. A 30-y study of risk of dairy fats on type 2 diabetes in a cohort of Swedish adults reported that cream and butter intake were inversely related to the disease [21]. However, studies investigating the effects of long-term consumption of full-fat dairy on cardiometabolic health measures in metabolically healthy overweight and obese adults remain limited.

Therefore, the objective of this study was to describe the effect of regular consumption of 3 servings of full-fat dairy for 12 wk in an energy-neutral and ad libitum diet compared with an energy-restricted diet by healthy overweight and obese adults while counseled to follow CFG. We hypothesized that adding 3 servings of full-fat dairy combined with counseling to follow the CFG would not adversely affect cardiometabolic biomarkers but would increase intake of limiting nutrients and decrease intake of food and beverages associated with chronic diseases.

## Methods

### Study design

A single-blinded, randomized, parallel, multisite study was conducted at the University of Toronto (UofT) and Mount Saint Vincent University (MSVU). Block randomization was performed prior to recruitment on SAS version 9.4 (SAS Institute Inc.) by the study dietitian to generate a random allocation sequence stratified by sex with a block size of 12. Recruited participants were blinded to the dietary interventions and assigned to the next treatment group allocation in the sequence. Male and female participants (*n* = 74 total) were randomized to 1 of the 3 diet intervention groups for 12 wk: low dairy energy restriction diet (LD-ER), 3-dairy energy-neutral diet (3D-EN), and 3-dairy ad libitum diet (3D-AL). A registered dietitian counseled participants in the LD-ER to reduce their daily caloric intake by 500 kcal and to limit their consumption of dairy products to < 1 serving per day, choosing low-fat dairy options or plant-based alternatives. Participants in the 3D-EN arm were counseled to add 3 servings of full-fat dairy to their daily diet, which was reduced by 500 kcal to be energy neutral. The 3D-AL group consumed 3 daily servings of full-fat dairy and received no advice about their caloric intake. The novelty of the design rested with the concurrent counseling of participants to adjust their diet to align with the 2019 CFG.

Participants attended biweekly study visits. At weeks 0 (baseline) and 12, all measures were taken including baseline and physical activity questionnaires, blood pressure, BW, height, WC, hip circumference (HC), body composition, blood sample, urine sample, resting metabolic rate (RMR), dietary history, food record, Knowledge, Attitudes and Practices (KAP) Questionnaire, and dairy log. At weeks 2, 6, and 10, baseline questionnaires, weight measurement, and dairy logs were completed. At weeks 4 and 8, a physical activity questionnaire and blood and urine samples were collected. Three-day food records were completed at weeks 0, 4, and 8, whereas the food tracker was completed at weeks 2, 6, 10, and 12. Dietary counseling was provided at each visit with more in-depth sessions at weeks 0, 4, and 8 (Table 1). The experimental procedures were reviewed and approved by the Human Subject Review Committee at the UofT Ethics Review Office and the University Research Ethics Board at MSVU in Halifax. This study is registered on ClinicalTrials.gov as NCT04399460.

**TABLE 1:** Study protocol completion timepoints

	Week						
	0	2	4	6	8	10	12
Completed during study sessions							
Baseline questionnaire	•	•	•	•	•	•	•
Physical activity questionnaire	•		•		•		•
Blood pressure	•						•
Weight	•	•	•	•	•	•	•
Height	•						•
Finger-prick blood glucose sample	•		•		•		•
Venous blood sample	•		•		•		•
Spot urine sample	•		•		•		•
Waist and hip circumference	•						•
BOD POD	•						•
Metabolic cart	•						•
Dietary history questionnaire II	•						•
Knowledge, attitudes, practices questionnaire	•						•
Nutrition counseling							
30–40 min	•		•		•		
10 min		•		•		•	
Completed between study sessions							
3-d food record	•		•		•		
Food tracker		•		•		•	•
Dairy log^1^	•	•	•	•	•	•	•

Abbreviations: 3D-AL, 3-dairy ad libitum; 3D-EN, 3-dairy energy neutral.

1 Only completed by participants in the 3D-EN and 3D-AL groups.

### Participants

Healthy overweight to obese male and female adults aged between 25 and 60 y with a BMI (in kg/m^2^) between 25 and 34.9 were recruited through advertisements placed on the UofT and MSVU campuses, the Toronto Transit Commission subway, and online platforms including Reddit, Kijiji, and Facebook. Exclusion criteria included WC <88 cm for females or <102 cm for males, blood pressure >140/90 mmHg, fasting blood glucose (BG) >7 mmol/L, self-reported gastrointestinal symptoms to dairy, history of chronic illness or cardiometabolic disease, pregnant or lactating, menopausal or postmenopausal females, taking medications or supplements that would affect outcome measures, smokers, marijuana use more frequent than 1–2 times a month, and a history of consistent dieting.

The original sample size for this study, based on BW as the primary dependent measure, was 153 participants. This sample size was calculated with 124 participants being required to detect a difference of 2 kg change in BW between treatment groups with an estimated power level of 0.80 and an α = 0.05, and accounting for a 15%–20% dropout rate. Unfortunately, delays due to the COVID-19 pandemic allowed only 107 participants to be recruited and 74 to be completed for this study. Participant follow-up was conducted on an ongoing basis during in-person study sessions and between the weeks via email to ensure study compliance and their well-being. Participants were withdrawn from the study if they reported discomfort or distress with components of the study protocol.

### Treatments

The dairy products used in the study were Neilson TruTaste Microfiltered Homogenized Milk (3.25% milk fat (MF), Saputo Inc.), Danone Oîkos Greek Yogurt in assorted flavors (2% MF, Danone), and Armstrong Cheese Sticks in assorted flavors (31% MF, Saputo Inc.). Nutritional facts for the products are shown in Table 2. These products were selected based on fat content and availability in the marketplace. The initial yogurt selected for this study, Liberté Greek Yogurt with 35% less sugar (3% MF, Liberté Inc.), was discontinued due to COVID-19. Three participants completed the remainder of the study with the Danone yogurt, which was similar in nutrient content. All dairy was purchased from the marketplace and provided to the participants during their study visits.

**TABLE 2:** Nutrient composition of dairy treatments

	Treatments^1^													
	Milk^2^	Cheese sticks^3^		Previous yogurt^4^	Yogurt^5^									
		A	B		A	B	C	D	E	F	G	H	I	J
Weight (g)	258^6^	21	21	100	100	100	100	100	100	100	100	100	100	100
Energy (kcal)	160	80	80	80	70	80	90	90	90	90	90	90	90	90
Total fat (g)	8	7	7	2.5	2	1.5	1.5	1.5	1.5	1.5	1.5	1.5	1.5	1.5
Saturated fat (g)	5	4.5	4.5	1.5	1.5	1	1	1	1	1	1	1	1	1
Trans fat (g)	0.2	0.2	0.2	0.1	0	0	0	0	0	0	0	0	0	0
Cholesterol (mg)	30	20	20	10	10	10	10	10	10	10	10	10	10	10
Sodium (mg)	125	130	150	35	30	25	25	30	25	30	35	30	40	50
Carbohydrates (g)	12	1	1	7	4	9	11	11	11	11	11	12	12	12
Fiber (g)	0	0	0	0	0	0	0	0	0	0	0	0	0	0
Sugars (g)	12	0	0	6	3	7	9	9	10	10	10	10	10	10
Protein (g)	8	5	5	8	9	8	8	8	8	8	8	8	8	8
Calcium (mg)	330	110	110	88	100	75	75	75	75	75	75	75	75	75

1 Data provided by manufacturer from nutrition facts table on packaging.

2 Neilson TruTaste Microfiltered Homogenized Milk (3.25% MF), Saputo Inc.

3 Armstrong Cheese Sticks (31% MF), Saputo Inc. A: Garden Herbs and Old Cheddar flavors. B: Marble Cheddar flavor.

4 Liberté Greek Yogurt at 35% less sugar (3% MF), Mango, Raspberry, and Vanilla flavor, Liberté Inc. Discontinued during the study, as of spring 2022.

5 Danone Oîkos Greek Yogurt (2% MF), Danone, Boucherville, Quebec, Canada. Replacement yogurt used in the study, as of spring 2022. A: Plain flavor; B: Blueberry flavor; C: Banana, Blackberry, and Vanilla flavor; D: Strawberry flavor; E: Strawberry Banana flavor; F: Honey, Pineapple, and Strawberry Raspberry flavor; G: Key Lime flavor; H: Raspberry Pomegranate flavor; I: Mandarin Orange flavor; J: Passion Fruit.

6 Weight based on 250 mL of milk.

Participants were instructed to consume 250 mL of milk at breakfast with a serving of carbohydrates, one 100 g container of yogurt at lunch, and two 21 g cheese sticks, totaling 42 g of cheese, at dinner. They were advised to consume yogurt and cheese 7–10 min before a meal so that the first-phase insulin response would be present at the beginning of the meal. The dairy serving sizes were based on Health Canada’s Reference Amounts of 250 mL for milk, 125 g for yogurt, and 30 g for cheese [22]. Two packages of cheese sticks provide similar protein (10 g) to the servings of milk and yogurt at 8 g. Participants were allowed to switch the order of yogurt and cheese consumption.

### Experimental protocol

Participants arrived for their on-site visits between 08:00 and 10:00 AM after a 12-h overnight fast with water allowed ≤1 h before the visit. No strenuous physical activity or alcohol consumption was allowed 24 h before. On arrival, participants completed questionnaires to assess the consistency of their activities for the past 24 h and over the past month, including sleep, stress, alcohol consumption, and the previous day's food intake. The Canadian Society for Exercise Physiology - Physical Activity Training for Health (CSEP-PATH) Physical Activity and Sedentary Behaviour Questionnaire was used to assess physical activity [23,24]. During the COVID-19 pandemic, virtual sessions were held when in-person visits were not possible.

BG was measured on arrival through a finger-prick sample using a handheld glucometer (Accu-Chek Aviva; Roche Diagnostics Canada) to ensure that the participant was fasted. Intravenous blood samples were collected into 4 mL BD Vacutainer K_2_EDTA tubes (BD Diagnostics) at weeks 0 and 12 for HbA1c analysis, as well as 5 mL BD Vacutainer SST II *Advance* tubes (BD Diagnostics) at weeks 0, 4, 8, and 12. The SST tube sample was allowed to clot before being centrifuged at 3600 ×*g* for 10 min at 4°C (Thermo Electron Corporation). A 500 μL serum sample was aliquoted into Eppendorf tubes for analysis of total cholesterol (TC), HDL cholesterol, non-HDL cholesterol, LDL cholesterol, and TG, and stored at −80°C. A spot urine sample was also collected during the week 0, 4, 8, and 12 study visits, with 1500 μL aliquoted into an Eppendorf tube for analysis of creatinine, urea, and a measure of protein intake. Samples collected at MSVU were frozen at −80°C and sent to UofT for storage and analysis. The blood and urine samples were analyzed by the Pathology and Laboratory Medicine Department at Mount Sinai Hospital via clinical analyzer (Roche Diagnostics Canada).

WC was measured at the top of the iliac crest, and HC was measured at the maximum extension of the buttocks. Densitometry was measured via BOD POD (COSMED USA Inc). Body composition values including percent fat mass (FM) and fat-free mass (FFM) were calculated using the Siri equation in the BOD POD program. RMR was measured via a metabolic cart (ParvoMedics Inc). The measurement was 30 min long, with participants kept awake in a quiet, sedentary state for the duration. The first 10 min of the measurement were excluded from the analysis to allow for stabilization of the measures.

Participants completed the Dietary History Questionnaire (DHQ) II, created by the United States National Cancer Institute, to indicate their past month’s diet. A modified version was used, based on the Canadian DHQ-II and updates from the American DHQ-III of 2018 [25,26]. The KAP questionnaire, designed following guidelines of the FAO, was used to obtain understanding and thoughts toward nutrition and the CFG [27]. Participants in the 3D-EN and 3D-AL groups also completed a dairy log every 2 wk in which they documented the product flavor, time of consumption, and time of lunch and dinner to assess adherence with the dairy intervention.

Nutrition counseling was provided for 30 min at weeks 4 and 8, and for 10 min at weeks 2, 6, and 10. An explicit goal of the counseling was to encourage participants to utilize the CFG as their dietary guidance. Participants also completed 3-d food records and food trackers over the 12-wk and were taught how to use these tools by the study dietitian. These assessment tools provided information about the participants’ eating patterns and adherence to the CFG recommendations [28]. The dietitian provided tailored guidance to adjust their diets to the study protocol.

Three-day food records were completed on weeks 0, 4, and 8 to assess nutrient intake. The participants recorded the amounts of all foods, snacks, and beverages consumed over 3 d, which included 2 nonconsecutive weekdays and 1 weekend. Participants were instructed to be specific when recording the type of foods or beverages consumed and to include all parts of what was eaten, including sauces and seasonings. Measuring cups and spoons were provided, and guidance to use a scale or the hand serving size guide was provided to help with estimating food amount [29]. Participants were also instructed to use standard measurement units (g, mL, cups, tbsp, etc.) and specify the cooking method, such as whether the food was raw, grilled, or fried. For products purchased or foods prepared at a restaurant, information about brand name, product type (e.g., “low fat,” “low sodium,” “sugar free”), restaurant name, and menu items were to be included.

The food trackers were collected on weeks 2, 6, 10, and 12 to assess food intake. These were simplified food records that documented participant intake by serving size for food categories determined based on the Healthy Eating Food Index and recommendations of the CFG [19,30,31]. All foods and drinks consumed were tracked for ≥7 d, which did not have to be consecutive but included ≥2 weekend days. Participants recorded the number of servings in the appropriate category on the tracker. If a food item fit into multiple categories, it was listed in all of them. For example, a serving of salmon was recorded as a healthy fat and an animal-based protein. Complex foods were broken down into their main ingredients, such that a bowl of chicken noodle soup would be vegetables, chicken, white pasta, and butter. Seasonings and sauces were not tracked unless used in large quantities. Food skills, including reading food labels, cooking at home, and using healthy cooking methods, were tracked, as well as the participant’s frequency of dining out. A table categorizing various foods and a hand serving size guide were provided to help participants [29].

### Data analysis

Statistical analysis was conducted using SAS version 9.4. Three-way Analysis of variance (ANOVA) was used to determine treatment, week, and sex effects on the dependent measures. Including sex as a factor ensured that potential variability due to sex was accounted for, whereas interpretation focused on treatment, week, and their interaction. Two-way ANOVA was used to determine treatment and sex differences in mean values. When sex was not a factor, it was removed from the statistical models. For assessing changes in outcome measures from weeks 0 to 12, a paired or 1-sample *t*-test was used to determine the change within each treatment group. This was followed by a 1-way ANOVA to compare the effect of treatment on the changes from baseline among the groups. KAP questionnaire responses provided on a scale of 0 (least) to 10 (most) were averaged for mean values, whereas yes/no answers were tallied for qualitative questions. A paired t-test was used to compare week 0 and week 12 responses. Dairy logs were analyzed for treatment group and sex effects on compliance using 2-way ANOVA. Food intake and weekly food skills usage were assessed based on data tabulated from the food trackers. Nutrient intake was calculated based on analysis of the 3-d food records obtained at baseline and weeks 4 and 8 using Cronometer (Cronometer Software Inc.). Nutrient intakes were calculated for protein, fat, calcium, magnesium, potassium, vitamins A, B2, B12, D, and total energy. Reported dietary intake was analyzed using 3-way ANOVA to determine the effects of treatment, time, and sex. Two-way ANOVA was used to determine treatment and sex differences in averaged dietary intakes. Tukey-Kramer post hoc test was used to identify pairwise differences, with *P* value of <0.05 used to determine statistical significance.

## Results

### Participant characteristics

**FIGURE 1:**
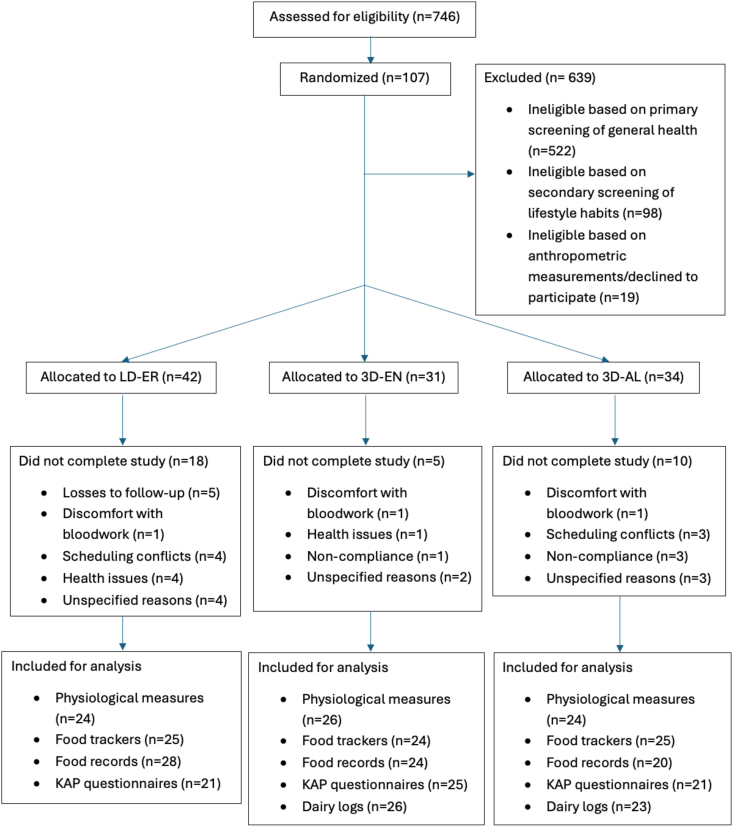
Flow diagram of study participants. Abbreviations: 3D-AL, 3 dairy ad libitum; 3D-EN, 3 dairy energy neutral; KAP, Knowledge Attitudes and Practices Questionnaire; LD-ER, low dairy energy restriction.

Data collection was conducted from September 2020 to February 2023. Overall, 746 individuals were screened for eligibility. A total of 107 participants were enrolled, of which 74 participants completed the study from UofT (*n* = 43) and from MSVU (*n* = 31). The remaining 33 participants could not complete the entire study due to reasons including losses to follow-up, scheduling conflicts, discomfort with bloodwork, health issues, and noncompliance. However, data collected from the withdrawn participants were included if baseline data were available for the assessment of change. Missing data also required adjustments in the sample size of analysis for some outcome measures, outlined in Figure 1. The number of males and females was not evenly distributed.

Participants were 36.55 ± 1.04 y old with a BMI of 29.34 ± 0.43. At baseline, glycemia (BG: 5.43 ± 0.07 mmol/L; HbA1c: 5.33% ± 0.04%), cholesterol (total: 5.07 ± 0.11 mmol/L; LDL: 3.10 ± 0.10 mmol/L; HDL: 1.37 ± 0.04 mmol/L; non-HDL: 3.71 ± 0.11 mmol/L), TG (1.34 ± 0.08 mmol/L), and blood pressure (systolic: 118.77 ± 1.23 mmHg; diastolic: 72.55 ± 1.17 mmHg) were within clinically normal ranges. The baseline measurements were similar among treatment groups (*P* > 0.20), but a sex difference was found. Males had higher baseline BW, height, BMI, blood pressure, waist-hip-ratio (WHR), FFM, RMR, and non-HDL cholesterol. Females had higher baseline HDL cholesterol and FM. Results are presented as means ± SEM (Table 3).

**TABLE 3:** Baseline (week 0) characteristics of participants by sex

Measure	Treatment groups						*P*		
	LD-ER (*n* = 24)		3D-EN (*n* = 26)		3D-AL (*n* = 24)		Treatment	Sex	Treatment × sex
	Male (*n* = 11)	Female (*n* = 13)	Male (*n* = 11)	Female (*n* = 15)	Male (*n* = 12)	Female (*n* = 12)			
Age (y)	35.45 ± 3.22	40.62 ± 2.11	38.27 ± 3.17	35.00 ± 1.80	34.75 ± 2.53	35.33 ± 2.73	0.5151	0.6951	0.2621
Weight (kg)	103.05 ± 5.47^a^	72.36 ± 1.78^b^	91.35 ± 3.17^a^	83.02 ± 2.86^b^	91.16 ± 3.57^a^	76.24 ± 2.79^b^	0.4380	<0.0001	0.0044
Height (cm)	179.59 ± 1.70	162.16 ± 1.44	177.71 ± 2.43	165.43 ± 1.31	178.66 ± 1.81	162.16 ± 2.13	0.8079	<0.0001	0.3132
BMI (kg/m^2^)	31.90 ± 1.72^a^	27.52 ± 0.81^b^	28.94 ± 0.88^ab^	30.22 ± 0.84^ab^	28.43 ± 0.73^ab^	29.11 ± 0.98^ab^	0.6133	0.3353	0.0131
WC (cm)	105.56 ± 3.56	94.26 ± 2.73	101.34 ± 2.65	100.46 ± 3.01	100.38 ± 2.41	98.18 ± 3.86	0.8692	0.0624	0.1976
HC (cm)	110.34 ± 3.20	106.14 ± 1.24	105.21 ± 1.17	111.36 ± 1.74	104.71 ± 1.74	105.61 ± 2.04	0.1896	0.5493	0.0323
WHR	0.96 ± 0.02	0.89 ± 0.02	0.96 ± 0.02	0.90 ± 0.02	0.96 ± 0.01	0.93 ± 0.02	0.5681	0.0011	0.5862
WHtR	0.59 ± 0.02	0.58 ± 0.02	0.57 ± 0.02	0.61 ± 0.02	0.56 ± 0.01	0.61 ± 0.03	0.9708	0.1138	0.3615
FFM (%)	68.67 ± 2.71	61.77 ± 1.71	69.51 ± 2.42	58.19 ± 1.22	69.18 ± 2.34	60.65 ± 1.83	0.7711	<0.0001	0.5389
FM (%)	31.33 ± 2.71	38.23 ± 1.71	30.49 ± 2.42	41.81 ± 1.22	30.83 ± 2.34	39.35 ± 1.83	0.7711	<0.0001	0.5389
RMR (kcal/d)	2098.88 ± 68.79	1356.39 ± 50.95	1886.34 ± 99.57	1494.11 ± 73.01	1930.36 ± 81.56	1365.98 ± 65.97	0.5798	<0.0001	0.0721
SBP (mmHg)	128.21 ± 2.58	112.36 ± 2.49	121.24 ± 2.44	114.53 ± 1.87	124.89 ± 2.80	113.94 ± 3.35	0.6451	<0.0001	0.2182
DBP (mmHg)	77.64 ± 3.37	68.21 ± 2.45	74.15 ± 2.80	70.87 ± 2.33	75.56 ± 2.42	70.19 ± 3.58	0.9872	0.0113	0.5467
BG (mmol/L)	5.50 ± 0.14	5.34 ± 0.17	5.45 ± 0.19	5.57 ± 0.16	5.24 ± 0.11	5.45 ± 0.19	0.6161	0.6621	0.4975
TC (mmol/L)	4.86 ± 0.32	4.94 ± 0.23	5.54 ± 0.32	4.80 ± 0.22	5.14 ± 0.21	5.26 ± 0.34	0.4997	0.4221	0.2185
HDL-C (mmol/L)	1.20 ± 0.09	1.61 ± 0.14	1.30 ± 0.07	1.38 ± 0.07	1.19 ± 0.08	1.49 ± 0.08	0.7379	0.0011	0.1867
Non-HDL-C (mmol/L)	3.66 ± 0.29	3.33 ± 0.28	4.25 ± 0.31	3.42 ± 0.22	3.96 ± 0.23	3.76 ± 0.32	0.3483	0.0464	0.4745
LDL-C (mmol/L)	3.08 ± 0.27	2.88 ± 0.25	3.53 ± 0.27	2.77 ± 0.18	3.23 ± 0.16	3.24 ± 0.30	0.5604	0.1116	0.2638
TG (mmol/L)	1.3 ± 0.13	0.99 ± 0.14	1.55 ± 0.28	1.44 ± 0.18	1.63 ± 0.24	1.18 ± 0.08	0.1585	0.0600	0.6403
HbA1c (%)	5.33 ± 0.10	5.31 ± 0.06	5.30 ± 0.11	5.32 ± 0.10	5.46 ± 0.07	5.28 ± 0.09	0.7883	0.4148	0.5147
Creatinine (mmol/L)	12.02 ± 2.05	11.98 ± 2.46	20.05 ± 3.88	11.23± 1.80	12.34 ± 2.17	19.24 ± 2.92	0.2603	0.7586	0.0118
Urea (mmol/L/kg)	2.78 ± 0.51^ab^	2.97 ± 0.46^ab^	3.34 ± 0.57^ab^	3.03 ± 0.46^ab^	2.47 ± 0.42^a^	4.51 ± 0.49^b^	0.4330	0.1032	0.0404
UCR (mmol/L/mmol/L)	23.40 ± 1.77	22.50 ± 2.41	18.07 ± 2.23	23.58 ± 1.42	19.53 ± 1.37	20.92 ± 2.48	0.3661	0.2225	0.2664
PIUR (g/mmol/L)^1^	0.72 ± 0.10	0.74 ± 0.20	0.54 ± 0.08	0.37 ± 0.05	0.66 ± 0.10	0.17 ± 0.01	0.3291	0.3087	0.5645

Data are presented as baseline means for each sex (means ± SEM; *n* = 74). Two-way ANOVA analysis for baseline (week 0) measures with treatment (*P >* 0.20) and sex (*P* < 0.01) as independent factors. Statistical significance determined using Tukey-Kramer post hoc test (*P* < 0.05). Different letters within each row denote values with significant differences.

Abbreviations: ANOVA, Analysis of variance; 3D-AL, 3 dairy ad libitum; 3D-EN, 3 dairy energy neutral; BG, blood glucose; DBP, diastolic blood pressure; FFM, fat-free mass; FM, fat mass; HbA1c, hemoglobin A1c; HC, hip circumference; LD-ER, low dairy energy restriction; PIUR, protein intake-urea excretion ratio; RMR, resting metabolic rate; SBP, systolic blood pressure; TC, total cholesterol; TG, triglyceride; UCR, urea-creatinine ratio; WC, waist circumference; WHR, waist-hip ratio; WHtR, waist-height ratio.

1 PIUR was calculated for *n* = 35.

### Anthropometric measures

**FIGURE 2:**
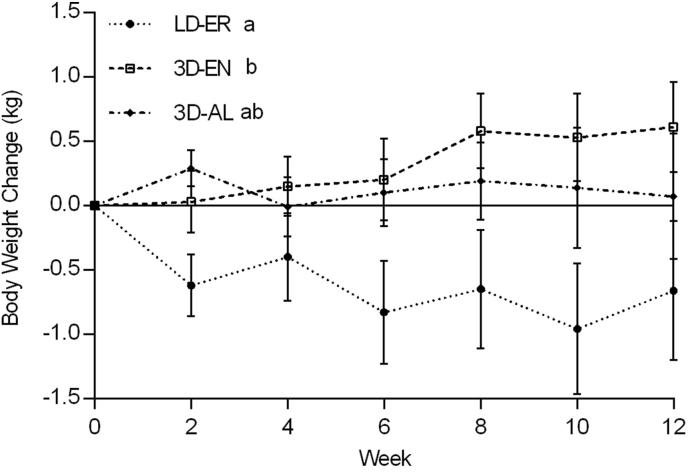
Change in body weight (kg) over 12 wk. Data are means of change from baseline ± SEM (*n* = 74). Three-way ANOVA with treatment, week, and sex as independent factors. Tukey-Kramer post hoc test (*P <* 0.05) used to detect significant differences as shown by letter superscripts. Body weight was affected by treatment (*P =* 0.039) but not week (*P =* 0.88) or treatment-by-week interaction (*P =* 0.07) over the 12 wk. The interaction approached statistical significance because there was a 0.35 ± 0.25 kg increase in the 3D-EN compared with a 0.69 ± 0.37 kg decrease in LD-ER (*P <* 0.04) group. The change in 3D-AL was a 0.14 ± 0.27 kg increase, which was not significantly different from the other treatment groups. Tukey-Kramer post hoc test, *P <* 0.05. Abbreviations: ANOVA, Analysis of variance; 3D-AL, 3 dairy ad libitum; 3D-EN, 3 dairy energy neutral; LD-ER, low dairy energy restriction.

There was a treatment effect found for BW (*P* = 0.0064) but not time (*P* = 0.92) or treatment-by-time interaction (*P* = 0.07) effects over the 12 wk (Table 4). The interaction approached statistical significance because there was a 0.35 ± 0.25 kg increase in the 3D-EN compared with a 0.69 ± 0.37 kg decrease in LD-ER (*P* < 0.04) group with a 95% confidence interval (CI): –2.1, –0.06, regardless of time. The change in 3D-AL was a 0.14 ± 0.27 kg increase, which was not significantly different from the other treatment groups (Table 4; Figure 2). A treatment effect (*P* = 0.0061) was found for BMI, but no week (*P* = 0.93) or treatment-by-week interaction effects (*P* = 0.09) were detected. Over 12 wk, the decrease in BMI by –0.22 ± 0.12 in LD-ER was different from the increase of 0.10 ± 0.08 in 3D-EN (*P* = 0.047; 95% CI: –0.7, –0.004), but not from the 0.03 ± 0.09 increase in 3D-AL (*P* = 0.18; 95% CI: –0.6, 0.09), regardless of time (Table 4). Week 0 and 12 measures of BW and BMI were not different between the treatment groups (*P* > 0.40). No differences between treatment groups (*P* > 0.60) or change from week 0 to 12 (*P* > 0.20) were found for WC, WHR, and waist-height ratio (WHtR; Table 5). However, HC was reduced from baseline by 0.25 ± 1.64 cm (*P* = 0.048; 95% CI: 0.008, 1.4) and systolic blood pressure (SBP) by 2.72 ± 2.18 mmHg (*P* = 0.04; 95% CI: 0.2, 7.3) in 3D-AL participants. In the LD-ER group, SBP was also reduced from baseline by 4.25 ± 2.20 mmHg (*P* = 0.049; 95% CI: 0.01, 7.6). No treatment group differences were observed for the changes in HC (*P* = 0.59) and SBP (*P* = 0.09). There were no time (*P* > 0.10) or treatment (*P* = 0.99) effects for diastolic blood pressure (DBP) (Table 5). Males had higher WHR by 0.04 ± 0.14 (*P* < 0.006), SBP by 9.58 ± 1.74 mmHg (*P* < 0.0001), and DBP by 5.31 ± 1.76 mmHg (*P* < 0.02) than females at week 12.

**TABLE 4:** Baseline and change from baseline (week 0) over 12 wk in physiological measurements

Measure	Treatment groups			*P*	Change from baseline				
	LD-ER (*n* = 24)	3D-EN (*n* = 26)	3D-AL (*n* = 24)		*P*				
					Treatment	Time	Sex	Treatment × time	Treatment × time × sex
Weight (kg)									
Baseline	86.43 ± 4.12	86.55 ± 2.24	83.70 ± 2.71	0.4380	0.0064	0.9202	0.2641	0.0750	0.6580
Mean change	–0.69 ± 0.37^a^	+0.35 ± 0.25^b^	+0.14 ± 0.27^ab^	0.0374					
Week 12	85.77 ± 4.25	87.16 ± 2.36	83.74 ± 2.94	0.4204					
BMI (kg/m^2^)									
Baseline	29.53 ± 0.99	29.68 ± 0.62	28.77 ± 0.60	0.6133	0.0061	0.9256	0.3336	0.0933	0.6909
Mean change	–0.22 ± 0.12^a^	+0.10 ± 0.08^b^	+0.03 ± 0.09^ab^	0.0496					
Week 12	29.26 ± 1.02	29.86 ± 0.64	28.81 ± 0.61	0.6629					
BG (mmol/L)									
Baseline	5.38 ± 0.11	5.52 ± 0.12	5.35 ± 0.11	0.5851	0.7006	0.7757	0.5311	0.7844	0.3973
Mean change	–0.04 ± 0.10	–0.08 ± 0.11	+0.03 ± 0.09	0.7805					
Week 12	5.36 ± 0.10	5.45 ± 0.10	5.40 ± 0.14	0.8496					
TC (mmol/L)									
Baseline	4.90 ± 0.20	5.11 ± 0.20	5.20 ± 0.20	0.4888	0.6731	0.4136	0.4469	0.9572	0.1344
Mean change	–0.06 ± 0.11	–0.11 ± 0.08	–0.01 ± 0.09	0.7215					
Week 12	4.77 ± 0.16	4.93 ± 0.22	5.08 ± 0.20	0.5387					
HDL-C (mmol/L)									
Baseline	1.44 ± 0.10	1.35 ± 0.05	1.34 ± 0.06	0.6797	0.7458	0.3475	0.9253	0.3203	0.3867
Mean change	–0.03 ± 0.03	–0.04 ± 0.02	–0.02 ± 0.03	0.7089					
Week 12	1.42 ± 0.09	1.33 ± 0.06	1.28 ± 0.06	0.5273					
LDL-C (mmol/L)									
Baseline	2.96 ± 0.19	3.09 ± 0.17	3.24 ± 0.17	0.5598	0.5352	0.6111	0.9147	0.9769	0.0304
Mean change	–0.04 ± 0.08	–0.09 ± 0.06	–0.03 ± 0.08	0.7530					
Week 12	2.85 ± 0.16	2.92 ± 0.17	3.17 ± 0.19	0.4664					
Non-HDL-C (mmol/L)									
Baseline	3.46 ± 0.21	3.77 ± 0.19	3.86 ± 0.20	0.3220	0.7707	0.3992	0.4254	0.9837	0.0719
Mean change	–0.03 ± 0.03	–0.06 ± 0.08	+0.02 ± 0.08	0.8469					
Week 12	3.33 ± 0.18	3.60 ± 0.20	3.80 ± 0.21	0.3121					
TG (mmol/L)									
Baseline	1.09 ± 0.10	1.48 ± 0.15	1.40 ± 0.13	0.1069	0.3987	0.0103	0.0326	0.5620	0.6510
Mean change	+0.02 ± 0.06	+0.06 ± 0.10	+0.04 ± 0.06^∗^	0.7722					
Week 12	1.09 ± 0.10	1.47 ± 0.19	1.39 ± 0.13	0.1202					
Creatinine (mmol/L)									
Baseline	12.33 ± 1.63	14.96 ± 2.09	15.79 ± 1.92	0.3519	0.6710	0.5135	0.6681	0.5279	0.0876
Mean change	+0.58 ± 1.22	+0.14 ± 1.66	–0.79 ± 1.43	0.7582					
Week 12	11.72 ± 1.83	15.75 ± 1.69	14.37 ± 2.06	0.3431					
Urea (mmol/L/kg)									
Baseline	2.98 ± 0.34	3.16 ± 0.35	3.52 ± 0.39	0.5554	0.7110	0.1311	0.9385	0.8550	0.6188
Mean change	+0.08 ± 0.27	+0.52 ± 0.33	+0.25 ± 0.26	0.5978					
Week 12	2.68 ± 0.28	3.71 ± 0.38	3.43 ± 0.46	0.1492					
UCR (mmol/L/mmol/L)									
Baseline	23.08 ±1.57	21.25 ± 1.34	20.22 ± 1.39	0.3221	0.0679	0.3545	0.2894	0.6650	0.8080
Mean change	–0.25 ± 1.10	+1.70 ± 1.21	+2.91 ± 1.04^∗^	0.1130					
Week 12	23.07 ± 1.76	22.75 ± 1.54	22.77 ± 1.94	0.9762					
PIUR (g/mmol/L)									
Baseline	0.69 ± 0.24	0.43 ± 0.08	0.44 ± 0.13	0.5250	0.0850	0.1328	0.4329	0.0695	0.1842
Mean change	–0.08 ± 0.24	+0.34 ± 0.17	+0.09 ± 0.10	0.2383					
Week 8^1^	0.64 ± 0.18	1.06 ± 0.38	0.70 ± 0.19	0.2227					

Data are presented as the mean baseline, change from baseline over 12 wk, and week 12 values (mean ± SEM; *n* = 74). One-way ANOVA for determining treatment differences in baseline (*P >* 0.10), mean change from baseline (*P* < 0.05), and week 12 measures (*P >* 0.10). Different letters within each row indicate means with significant treatment differences detected using Tukey-Kramer post hoc test, *P* < 0.05. One-sample *t*-test comparing mean change from baseline over 12 wk to the baseline of zero in each treatment group, with asterisks indicating a significant mean change from baseline (∗ *P <* 0.05; ∗∗ *P <* 0.001; ∗∗∗ *P* < 0.0001). Two-tailed paired *t*-test comparing baseline and week 12 measurements within each treatment group, no significant differences were found (*P* > 0.05). Three-way ANOVA to determine treatment (*P* > 0.006), time (*P* > 0.01), sex (*P* > 0.08), and their interaction (*P* > 0.03) effects on the changes from baseline, with Tukey-Kramer post hoc test to determine statistical differences (*P <* 0.05).

Abbreviations: ANOVA, Analysis of variance; 3D-AL, 3 dairy ad libitum; 3D-EN, 3 dairy energy neutral; BG, blood glucose; LD-ER, low dairy energy restriction; PIUR, protein intake-urea excretion ratio; TC, total cholesterol; TG, triglyceride; UCR, urea-creatinine ratio.

1 Final PIUR was calculated at week 8.

**TABLE 5:** Comparison of physiological measures assessed at week 0 (baseline) and 12

Measure	Treatment groups									Change from baseline		
	LD-ER (*n* = 24)			3D-EN (*n* = 26)			3D-AL (*n* = 24)			*P*		
	Week 0	Week 12	*P*	Week 0	Week 12	*P*	Week 0	Week 12	*P*	Treatment	Sex	Treatment × sex
WC (cm)	99.44 ± 2.45	98.24 ± 2.56	0.2649	100.83 ± 2.03	100.49 ± 1.96	0.6390	99.28 ± 2.24	99.33 ± 2.40	0.3350	0.8733	0.6538	0.6341
HC (cm)	108.06 ± 1.63	107.82 ± 1.81	0.7220	108.76 ± 1.26	108.22 ± 1.19	0.1641	105.16 ± 1.31	104.91 ± 1.38	0.0478	0.5928	0.2047	0.4095
WHR	0.92 ± 0.01	0.91 ± 0.01	0.3215	0.93 ± 0.02	0.93 ± 0.01	0.8647	0.94 ± 0.01	0.95 ± 0.01	0.9182	0.6142	0.2318	0.7270
WHtR	0.58 ± 0.01	0.58 ± 0.02	0.3084	0.59 ± 0.01	0.59 ± 0.01	0.6371	0.58 ± 0.01	0.58 ± 0.02	0.3336	0.8972	0.6757	0.6727
FFM (%)	64.77 ± 1.66	64.90 ± 1.64	0.8591	62.98 ± 1.65	62.83 ± 1.68	0.1423	64.91 ± 1.70	64.85 ± 1.89	0.9208	0.9484	0.0718	0.1812
FM (%)	35.23 ± 1.66	35.10 ± 1.64	0.8591	37.02 ± 1.65	37.17 ± 1.68	0.1423	35.09 ± 1.70	35.15 ± 1.89	0.9208	0.9484	0.0718	0.1812
RMR (kcal/d)	1707.57 ± 89.19	1692.38 ± 92.12	0.9536	1641.51 ± 64.87	1671.43 ± 67.17	0.5209	1571.86 ± 101.96	1612.11 ± 107.16	0.7394	0.4657	0.5606	0.8933
SBP (mmHg)	119.63 ± 2.41	115.38 ± 2.45	0.0494	117.37 ± 1.61	118.46 ± 1.60	0.4642	119.42 ± 2.42	116.70 ± 2.33	0.0400	0.0905	0.9535	0.9480
DBP (mmHg)	72.53 ± 2.22	70.67 ± 1.91	0.2153	72.26 ± 1.79	70.31 ± 1.92	0.1127	72.88 ± 2.19	71.86 ± 2.20	0.4624	0.9911	0.3542	0.3930
HbA1C (%)	5.32 ± 0.06	5.27 ± 0.06	0.2942	5.31 ± 0.07	5.30 ± 0.07	0.7114	5.37 ± 0.06	5.37 ± 0.07	0.8272	0.9795	0.3643	0.3958

Data are presented as the mean week 0 and week 12 values (means ± SEM; *n* = 74). Two-tailed paired *t*-test analysis comparing week 0 and week 12 measurements within each treatment group, *P <* 0.05 denoting significant differences. Two-way ANOVA to determine treatment (*P* > 0.09), sex (*P* > 0.07), and their interaction (*P* > 0.10) effects on the change from baseline, and Tukey-Kramer post hoc test to determine statistical differences (*P <* 0.05).

Abbreviations: ANOVA, Analysis of variance; 3D-AL, 3 dairy ad libitum; 3D-EN, 3 dairy energy neutral; DBP, diastolic blood pressure; FFM, fat-free mass; FM, fat mass; HbA1c, hemoglobin A1c; HC, hip circumference; LD-ER, low dairy energy restriction; RMR, resting metabolic rate; SBP, systolic blood pressure; WC, waist circumference; WHR, waist-hip ratio; WHtR, waist-height ratio.

### Body composition and metabolic rate

There was no treatment (*P* > 0.40) or week 0 and 12 (*P* > 0.10) differences in FM, FFM, and RMR (Table 5). Males had higher FFM and lower FM at week 12 than females by 8.31% ± 1.58%, and higher RMR by 472.72 ± 10.85 kcal/d (*P* < 0.0001).

### Blood measures

**FIGURE 3:**
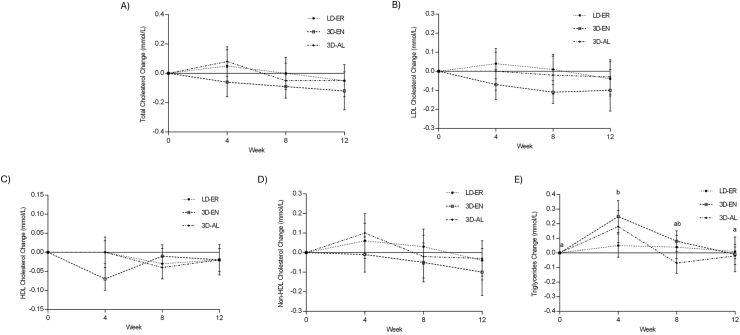
Change in blood lipids (mmol/L) over 12 wk. (A) Total cholesterol, (B) LDL cholesterol, (C) HDL cholesterol, (D) non-HDL cholesterol, and (E) triglycerides. Data are means of change from baseline ± SEM (*n* = 74). Three-way ANOVA with treatment, week, and sex as independent factors. There were no treatment group differences (*P >* 0.20), changes from baseline (*P >* 0.30), or treatment-by-week effects (*P >* 0.30) for total, LDL, HDL, and non-HDL cholesterols. There were triglyceride changes from baseline (*P =* 0.022), but no treatment group differences (*P =* 0.38) or interaction effects (*P =* 0.69). Tukey-Kramer post hoc test (*P <* 0.05) detected significant difference in changes from baseline as shown by letter superscripts. Abbreviations: ANOVA, Analysis of variance; 3D-AL, 3 dairy ad libitum; 3D-EN, 3 dairy energy neutral; LD-ER, low dairy energy restriction.

There were no treatment group differences (*P* > 0.50), changes from baseline (*P* > 0.30), or treatment-by-week effects (*P* > 0.30) for BG, LDL cholesterol, HDL cholesterol, non-HDL cholesterol, and TC (Table 4; Figure 3). No treatment group (*P =* 0.94) or week 0 and 12 differences (*P* > 0.20) were found for HbA1c (Table 5). TG changes from baseline (*P =* 0.01) existed, but no treatment group differences (*P =* 0.40) or interaction effects (*P =* 0.56) were found (Table 4; Figure 3). TG concentration increased from baseline at week 4 by 0.16 ± 0.06 mmol/L (*P =* 0.049; 95% CI: –0.3, –0.0004). This change was different from the 0.01 ± 0.05 mmol/L decrease from baseline at week 12 (*P =* 0.047; 95% CI: –0.3, –0.0001). In 3D-EN, males had 0.26 ± 0.35 mmol/L greater increase in TG (*P =* 0.018) and 0.08 ± 0.17 mmol/L greater decrease in HDL cholesterol (*P =* 0.030) than females. Mean TC in 3D-EN was also 0.69 ± 0.52 mmol/L higher for males than females (*P =* 0.018).

No treatment (*P >* 0.06), week (*P >* 0.10), or treatment-by-week (*P >* 0.06) differences were found for the changes from baseline in urinary creatinine, urea, urea-creatinine ratio, and protein intake-urea excretion ratio. The mean changes from baseline in each treatment group are presented in Table 4.

### Food intake

Data for the participants’ food and beverage intake are presented in Table 6. There were no treatment (*P =* 0.59), week (*P =* 0.96), or treatment-by-week (*P =* 0.53) effects in the changes from baseline in fruit and vegetable intake, but males had higher intakes than females by a mean of 0.35 ± 0.53 servings (*P =* 0.044). Increased whole-grain and decreased white and whole wheat intake occurred with time (*P <* 0.0001), but there were no treatment group differences (*P >* 0.90) or treatment-by-week effects (*P >* 0.10). Whole-grain consumption increased at all weeks in comparison to baseline by a mean of 0.38 ± 0.14 servings/d (*P <* 0.001), except at weeks 3–4 and 7–8. White and whole wheat food consumption decreased (*P <* 0.0001) by 2.7 ± 0.37 servings from baseline at the same weeks. Grain food intakes at weeks 3–4 and 7–8 were similar to week 0 and different from the other weeks (*P <* 0.008).

**TABLE 6:** Baseline and change from baseline (weeks 1–2) over 12 wk in daily dietary food and beverage intake by number of servings

Category	Treatment groups			*P*	Change from baseline				
	LD-ER (*n* = 25)	3D-EN (*n* = 24)	3D-AL (*n* = 25)		*P*				
					Treatment	Time	Sex	Treatment × time	Treatment × time × sex
Fruits and vegetables									
Baseline	3.56 ± 0.51	3.31 ± 0.39	3.19 ± 0.42	0.8358	0.5898	0.9624	0.0438	0.5287	0.1635
Mean change	+0.26 ± 0.53	–0.21 ± 0.55	–0.46 ± 0.38	0.5729					
Weeks 11–12	3.75 ± 0.31	3.40 ± 0.33	3.21 ± 0.35	0.5391					
Whole grains									
Baseline	0.53 ± 0.15	0.54 ± 0.17	0.40 ± 0.19	0.7902	0.9054	<0.0001	0.5494	0.2536	0.9821
Mean change	+0.31 ± 0.11^∗^	+0.22 ± 0.21	+0.21 ± 0.21	0.9263					
Weeks 11–12	1.06 ± 0.13^†^	0.66 ± 0.10	0.96 ± 0.21	0.1469					
White and whole wheats									
Baseline	4.24± 0.71	3.57 ± 0.32	4.48 ± 0.61	0.5108	0.9153	<0.0001	0.1736	0.1111	0.2175
Mean change	–2.11 ± 0.88^∗^	–1.64 ± 0.38^∗∗^	–2.13 ± 0.53^∗^	0.9263					
Weeks 11–12	1.26 ± 0.23^†^	1.37 ± 0.19^†††^	1.57 ± 0.22^††^	0.1469					
Plant proteins									
Baseline	0.36 ± 0.14	0.23 ± 0.09	0.65 ± 0.22	0.1848	0.3587	0.0550	0.6358	0.5698	0.0501
Mean change	+0.39 ± 0.13^∗^	+0.28 ± 0.14	+0.03 ± 0.22	0.3130					
Weeks 11–12	0.80 ± 0.15^†^	0.60 ± 0.13	0.59 ± 0.14	0.5000					
Animal proteins									
Baseline	2.99 ± 0.65	3.16 ± 0.55	2.93 ± 0.54	0.9589	0.5677	<0.0001	0.1098	0.1208	0.2454
Mean change	–1.24 ± 0.43^∗^	–1.17 ± 0.47^∗^	–0.81 ± 0.46	0.7714					
Weeks 11–12	1.79 ± 0.35^†^	1.30 ± 0.14^††^	1.82 ± 0.31	0.3005					
Ruminant meats									
Baseline	0.82 ± 0.48	0.48 ± 0.18	0.58 ± 0.21	0.7371	0.6327	0.0043	0.2793	0.8554	0.4209
Mean change	–0.50 ± 0.38	–0.23 ± 0.15	–0.39 ± 0.20	0.7613					
Weeks 11–12	0.32 ± 0.10	0.25 ± 0.07	0.29 ± 0.11	0.8777					
Dairy									
Baseline	0.87 ± 0.18	1.36 ± 0.22	1.40 ± 0.36	0.3197	0.0061	<0.0001	0.7656	0.0252	0.3619
Mean change	+0.44 ± 0.25 a	+1.60 ± 0.26 b ∗∗∗	+1.51 ± 0.33 b ∗∗	0.0107					
Weeks 11–12	1.25 ± 0.21^a^	2.92 ± 0.11 b^†††^	2.76 ± 0.24 ab^†^	<0.0001					
Healthy fats									
Baseline	2.00 ± 0.55	1.33 ± 0.31	1.68 ± 0.51	0.6170	0.7821	0.0006	0.5826	0.9699	0.8340
Mean change	–1.01 ± 0.58	–0.52 ± 0.33	–0.98 ± 0.51	0.7299					
Weeks 11–12	1.07 ± 0.17	0.83 ± 0.15	0.74 ± 0.18	0.3733					
Saturated fats									
Baseline	2.12 ± 0.91	1.08 ± 0.31	1.48 ± 0.59	0.5286	0.5415	<0.0001	0.2932	0.8101	0.1834
Mean change	–1.53 ± 0.88	–0.47 ± 0.32	–1.01 ± 0.57	0.5058					
Weeks 11–12	0.92 ± 0.25	0.54 ± 0.11	0.42 ± 0.09	0.0787					
Processed foods									
Baseline	0.92 ± 0.26	0.79 ± 0.15	0.85 ± 0.18	0.8981	0.6305	0.0041	0.8018	0.9649	0.5508
Mean change	–0.48 ± 0.26	–0.29 ± 0.21	–0.23 ± 0.20	0.7247					
Weeks 11–12	0.44 ± 0.12	0.39 ± 0.06	0.50 ± 0.15	0.7748					
Confectionary and baked goods									
Baseline	0.87 ± 0.24	0.81 ± 0.31	1.33 ± 0.59	0.6269	0.2365	0.0139	0.6528	0.1486	0.0713
Mean change	–0.24 ± 0.19	–0.11 ± 0.24	–0.87 ± 0.56	0.3211					
Weeks 11–12	0.61 ± 0.17	0.59 ± 0.11	0.39 ± 0.09	0.3653					
Water									
Baseline	3.20 ± 0.83	4.07 ± 1.02	3.68 ±1.09	0.8342	0.1876	0.9526	0.2643	0.4730	0.1856
Mean change	+1.31 ± 1.23	–1.27 ± 1.00	–0.02 ± 1.00	0.2597					
Weeks 11–12	5.91 ± 1.04^a^	3.32 ± 0.58^b^	4.37 ± 0.45^ab^	0.0402					
Unsweetened beverages									
Baseline	1.50 ± 0.36	1.96 ± 0.39	1.10 ± 0.28	0.2166	0.2430	0.0041	0.0807	0.0994	0.4554
Mean change	–0.73 ± 0.23^∗∗^	–0.68 ± 0.40	+0.03 ± 0.28	0.1701					
Weeks 11–12	0.67 ± 0.20	0.78 ± 0.22^†^	1.11 ± 0.26	0.3892					
Unsweetened plant-based beverages									
Baseline	0.21 ± 0.15	0.13 ± 0.11	0.10 ± 0.07	0.7853	0.8651	0.7197	0.3977	0.7931	0.9335
Mean change	+0.04 ± 0.11	+0.20 ± 0.23	+0.03 ± 0.05	0.6708					
Weeks 11–12	0.31 ± 0.10	0.14 ±0.14	0.26 ± 0.17	0.6885					
Sweetened beverages									
Baseline	0.68 ± 0.27	0.80 ± 0.30	0.79 ± 0.29	0.9477	0.6650	0.0350	0.0777	0.9600	0.7616
Mean change	–0.13 ± 0.20	–0.27 ± 0.24	–0.43 ± 0.30	0.7030					
Weeks 11–12	0.63 ± 0.17^a^	0.31 ± 0.08^ab†^	0.21 ± 0.06^b^	0.0261					
Alcohol									
Baseline	0.19 ± 0.11	0.38 ± 0.22	0.23 ± 0.12	0.6878	0.7916	0.0134	0.6362	0.0395	0.0996
Mean change	–0.05 ± 0.08	–0.18 ± 0.18	–0.13 ± 0.10	0.7588					
Weeks 11–12	0.28 ± 0.10	0.27 ± 0.08	0.17 ± 0.06	0.5915					

Data are presented as the mean baseline, change from baseline over 12 wk, and weeks 11–12 values (mean ± SEM; *n* = 74). One-way ANOVA for determining treatment differences in baseline (*P*>0.10), mean change from baseline (*P <* 0.02), and week 12 measures (*P <* 0.05). Different letters within each row indicate means with significant treatment differences detected using Tukey-Kramer post hoc test, *P <* 0.05. One-sample *t*-test comparing mean change from baseline over 12 wk to the baseline of zero in each treatment group, with asterisks indicating a significant mean change from baseline (∗ *P <* 0.05; ∗∗ *P <* 0.001; ∗∗∗ *P <* 0.0001). Two-tailed paired *t*-test comparing baseline and week 12 measurements within each treatment group, with daggers indicating a significant difference between week 12 and baseline values (^†^*P <* 0.05; ^††^*P <* 0.001; ^†††^*P <* 0.0001). Three-way ANOVA to determine treatment (*P* > 0.006), time (*P <* 0.97), sex (*P* > 0.04), and their interaction (*P* > 0.02) effects on the changes from baseline, with Tukey-Kramer post hoc test to determine statistical differences (*P <* 0.05).

Abbreviations: ANOVA, Analysis of variance; 3D-AL, 3 dairy ad libitum*;* 3D-EN, 3 dairy energy neutral; LD-ER, low dairy energy restriction.

Animal protein (*P <* 0.0001) and ruminant meat (*P =* 0.0043) intake decreased from baseline, but no treatment group (*P >* 0.50) or treatment-by-week effects (*P >* 0.10) existed. Daily consumption of animal protein foods decreased from week 0 by a mean of 1.68 ± 0.31 servings/d at weeks 1–2, 5–6, 9–10, and 11–12 (*P <* 0.0005). Ruminant meat intake was lowered by 0.53 ± 0.18 servings at week 1–2 (*P =* 0.0091) and by 0.43 ± 0.20 servings at week 11–12 (*P =* 0.044). No treatment (*P =* 0.36), week (*P =* 0.055), or treatment-by-week (*P =* 0.57) effects were observed for plant protein food consumption, but males had higher intake than females by 0.29 ± 0.28 servings (*P =* 0.029).

Dairy intake was affected by treatment (*P =* 0.0061), week (*P <* 0.0001), and treatment-by-week (*P =* 0.025) effects. There was a smaller average increase in dairy consumption in LD-ER (0.44 ±0.25 servings) than in 3D-EN (1.60 ± 0.26 servings; *P <* 0.0001) and 3D-AL (1.51 ± 0.33 servings; *P <* 0.001), which were similar. Overall, lower (*P <* 0.0001) amounts of dairy foods were consumed in LD-ER (1.20 ± 0.08 servings) than 3D-EN (2.78 ± 0.09 servings) and 3D-AL (2.64 ± 0.11 servings). Consumption of dairy foods increased at all weeks in 3D-EN (*P <* 0.005) and 3D-AL (*P <* 0.002). There was a higher increase from baseline in 3D-EN than LD-ER at weeks 3–4, 5–6, 9–10, and 11–12 by a mean of 1.39 ± 0.80 servings (*P <* 0.04). The change from baseline was also higher by 0.97 ± 0.74 servings at week 5+6 in 3D-AL than LD-ER (*P =* 0.035).

Healthy fat foods decreased from week 0 by a mean of 0.82 ± 0.31 servings (*P =* 0.0006), and intake of saturated fat foods decreased by 0.91 ± 0.38 servings (*P <* 0.0001) at all weeks except weeks 3–4. No treatment group (*P* > 0.50) or treatment-by-week (*P* > 0.80) differences existed. Processed foods, confectionery, and baked goods decreased by week (*P =* 0.0041; *P =* 0.014), but no treatment (*P* > 0.20) or treatment-by-week interaction (*P* > 0.10) effects were found. For processed foods, there was a decrease in daily intake at weeks 9–10 by 0.42 ± 0.15 servings (*P =* 0.021) and weeks 11–12 by 0.39 ± 0.14 servings (*P =* 0.031) compared with week 0. For confectionery foods and baked goods, consumption was lowered at weeks 9–10 by 0.54 ± 0.27 servings (*P =* 0.04) and weeks 11–12 by 0.56 ± 0.27 servings (*P =* 0.025) compared with baseline. Change in water, sweetened beverage, and unsweetened plant-based beverage consumption was not affected by treatment (*P* > 0.10), week (*P* > 0.05), or treatment-by-week interactions (*P* > 0.40). Unsweetened beverage intake decreased from week 0 at weeks 9−10 by 0.59 ± 0.23 servings/d (*P =* 0.019) and weeks 11–12 by 0.55 ± 0.26 servings/d (*P =* 0.037). There were significant changes from baseline (*P =* 0.0041) in unsweetened beverage intake, but no treatment group (*P =* 0.24) or treatment-by-week (*P =* 0.099) differences. Alcohol consumption decreased by 0.26 ± 0.12 servings/d from baseline (*P =* 0.0079) at weeks 7–8. In 3D-EN, the increase in consumption by 0.15 ± 0.23 servings at weeks 3–4 was significantly different from the decrease at weeks 7–8 by 0.37 ± 0.30 servings (*P =* 0.044). There were no treatment effects (*P =* 0.79) on the changes in alcohol intake, but significant week (*P =* 0.013) and treatment-by-week (*P <* 0.04) differences were found.

### Weekly food skills

Treatment group differences (*P* > 0.10), changes over time (*P* > 0.10), and treatment-by-week interaction effects (*P* > 0.09) were not found for the usage of food skills. On average, participants reported reading food labels 2.79 ± 0.33 times, eating out 2.46 ± 0.18 times, eating home-cooked meals 12.04 ± 0.49 times, and utilizing healthy cooking methods 9.22 ± 0.49 times each week (Table 7). There was a sex difference (*P =* 0.043) in the reading of food labels over the 12 wk, with males using them 0.48 ± 0.35 times fewer than at first assessment (weeks 1–2) and females using them 0.98 ± 0.36 times more.

**TABLE 7:** Mean number of times of weekly food skills usage and change from baseline (weeks 1–2) over 12 wk

Food skills	Treatment groups			*P*					
	LD-ER (*n* = 25)	3D-EN (*n* = 24)	3D-AL (*n* = 25)	Mean	Change from baseline				
				Treatment	Treatment	Time	Sex	Treatment × time	Treatment × time × sex
Using food labels									
Mean	3.38 ± 0.61	2.32 ± 0.35	2.71 ± 0.70	0.7295	0.1286	0.3011	0.0432	0.0939	0.8467
Mean change	+0.31 ± 0.59	–0.55 ± 0.32	+1.12 ± 0.39						
Eating out/getting takeout									
Mean	3.44 ± 0.42	1.94 ± 0.20	2.07 ± 0.27	0.0939	0.8525	0.9190	0.2191	0.9756	0.3672
Mean change	–0.10 ± 0.54	–0.26 ± 0.21	+0.12 ± 0.20						
Eating home-cooked meals									
Mean	10.45 ± 0.77	13.75 ± 0.79	11.82 ± 0.96	0.2234	0.6291	0.7456	0.9491	0.9091	0.2071
Mean change	+1.12 ± 0.75	–0.23 ± 0.66	+0.64 ± 0.90						
Using healthy cooking methods									
Mean	7.01 ± 0.74	11.10 ± 0.83	9.41 ± 0.91	0.2018	0.2819	0.1707	0.9734	0.5292	0.4870
Mean change	+1.84 ± 0.66	+0.05 ± 0.61	+0.60 ± 0.65						

Data are presented as the mean weekly usage and mean change from weeks 1 to 2 over 12 wk (mean ± SEM; *n* = 74). One-way ANOVA analysis with treatment (*P* > 0.09) as an independent factor to assess differences in mean weekly usage. Three-way ANOVA analysis to determine treatment (*P* > 0.10), time (*P* > 0.30), sex (*P* > 0.04), and their interaction (*P* > 0.09) effects on the changes from baseline. Tukey-Kramer post hoc test (*P <* 0.05) to determine statistical significance.

Abbreviations: ANOVA, Analysis of variance; 3D-AL, 3 dairy ad libitum; 3D-EN, 3 dairy energy neutral; LD-ER, low dairy energy restriction.

### Daily nutrient intake

Mean daily intake of nutrients is presented in Table 8 (*n =* 72). For energy, treatment (*P =* 0.0033) and treatment-by-week (*P =* 0.014) but not week (*P =* 0.36) differences were found for the changes from baseline. The change in energy intake was different between LD-ER and 3D-EN, with a mean 212.52 ± 82.96 kcal decrease in LD-ER compared with a 293.96 ± 97.19 kcal increase in 3D-EN (*P =* 0.0024). The mean change in 3D-AL by 86.17 ± 137.32 kcal was not different from the other groups. At week 8, energy intake decreased (*P =* 0.04) in LD-ER by 249.65 ± 116.56 kcal from baseline compared with 3D-EN, which increased (*P =* 0.0037) by 432.16 ± 131.69 kcal (*P =* 0.013). No differences were found at week 4. Average total daily energy intake was lower in LD-ER (1939.95 ± 64.36 kcal) than 3D-EN (2246.27 ± 78.87 kcal; *P <* 0.001) and 3D-AL (2162.41 ± 100.24 kcal; *P =* 0.016), which were similar. Males consumed 571.8 ± 10.95 kcal more calories each day than females (*P =* 0.0012).

**TABLE 8:** Baseline and changes from baseline (week 0) at weeks 4 and 8 in intakes of nutrients

Category	Treatment groups			*P*	Change from baseline				
	LD-ER (*n* = 28)	3D-EN (*n* = 24)	3D-AL (*n* = 20)		*P*				
					Treatment	Time	Sex	Treatment × time	Treatment × time × sex
Energy (kcal)									
Baseline	2060.21 ± 110.13	2068.03 ± 117.39	2138.23 ± 161.66	0.9011	0.0033	0.3560	0.7514	0.0144	0.0400
Week 4 change	–169.78 ±104.83	+140.21 ± 100.47	+42.65 ± 104.73	0.0888					
Week 8 change	–249.65 ± 116.56^a∗^	+432.16 ± 131.69^b∗^	+137.86 ± 278.46^ab^	0.0132					
Protein (g)									
Baseline	91.95 ± 5.90	91.39 ± 8.48	91.98 ± 10.75	0.9982	0.0587	0.0018	0.8114	0.0318	0.5901
Week 4 change	+2.43 ± 9.38	+8.95 ± 5.01	+10.29 ± 4.18^∗^	0.7389					
Week 8 change	–2.14 ± 9.42^a^	+35.04 ± 8.18^b∗^	+33.87 ± 20.79^ab^	0.0284					
Fat (g)									
Baseline	93.10 ± 10.27	87.09 ± 7.55	85.71 ± 8.30	0.8238	0.0844	0.0871	0.8478	0.0374	0.2412
Week 4 change	–1.25 ± 7.65	+3.80 ± 7.89	+7.79 ± 5.60	0.6920					
Week 8 change	–4.84 ± 10.60^a^	+31.14 ± 6.14^b∗∗∗^	+9.45 ± 12.10^ab^	0.0309					
Calcium (mg)									
Baseline	766.54 ± 72.01	924.87 ± 73.98	765.93 ± 70.07	0.2141	0.0009	0.0013	0.8536	0.0144	0.2348
Week 4 change	+4.59 ± 61.80^a^	+236.66 ± 70.41^b∗^	+371.74 ± 60.37^b∗∗∗^	0.0006					
Week 8 change	–47.67 ± 77.62^a^	+295.97 ± 87.00^ab∗^	+758.59 ± 417.01^b^	0.0265					
Magnesium (mg)									
Baseline	266.73 ± 23.87	266.21 ± 25.92	259.38 ± 32.56	0.9792	0.0808	0.1455	0.4392	0.2218	0.0300
Week 4 change	+0.16 ± 24.09	+66.04 ± 27.07^∗^	+17.70 ± 20.01	0.1416					
Week 8 change	+10.79 ± 35.11	+89.38 ± 43.84	+1.03 ± 36.22	0.2296					
Potassium (mg)									
Baseline	2431.51 ± 152.90	2441.60 ± 191.57	2519.16 ± 209.18	0.9383	0.1067	0.0619	0.4976	0.2711	0.2619
Week 4 change	+91.87 ± 196.16^a^	+639.90 ± 359.20^b^	+64.47 ± 183.37^a^	0.0025					
Week 8 change	–17.29 ± 192.16	+643.71 ± 252.21^∗^	+562.55 ± 532.84	0.2340					
Vitamin A (IU)									
Baseline	3911.58 ± 1128.66	5052.14 ± 971.19	7435.57 ± 2916.39	0.3478	0.7176	0.7600	0.7445	0.3711	0.0532
Week 4 change	+1126.79 ± 1715.97	–760.03 ± 1191.62	–2431.32 ± 2897.47	0.5479					
Week 8 change	–864.74 ± 1160.59	+709.88 ± 1690.50	–233.82 ± 1939.38	0.8715					
Vitamin B2 (mg)									
Baseline	1.76 ± 0.18	1.67 ± 0.15	1.65 ± 0.16	0.8660	0.3373	0.9860	0.2909	0.6218	0.5104
Week 4 change	–0.18 ± 0.19	+0.20 ± 0.17	+0.00 ± 0.21	0.3490					
Week 8 change	–0.20 ± 0.20	+0.16 ± 0.19	–0.01 ± 0.28	0.4639					
Vitamin B12 (μg)									
Baseline	5.23 ± 1.68	3.52 ± 0.42	4.27 ± 1.39	0.6422	0.9021	0.1509	0.6964	0.6289	0.6418
Week 4 change	–0.87 ± 1.84	+0.91 ± 0.64	–0.00 ± 1.49	0.6855					
Week 8 change	+3.95 ± 3.13	+1.38 ± 0.72	+1.27 ± 2.02	0.6540					
Vitamin D (IU)									
Baseline	247.10 ± 89.67	131.65 ± 21.23	147.33 ± 32.08	0.3573	0.1180	0.1954	0.5210	0.1747	0.3237
Week 4 change	–66.47 ± 97.48	+91.60 ± 32.51^∗^	+56.00 ± 41.99	0.2498					
Week 8 change	–79.81 ± 106.92^a^	+117.10 ± 51.58^ab∗^	+280.68 ± 229.06^b^	0.0013					

Data are presented as mean baseline and change from baseline values (mean ± SEM; *n* = 72). One-way ANOVA for determining treatment differences in baseline values (*P*>0.20) and mean changes from baseline at week 4 (*P <* 0.003) and week 8 (*P <* 0.04). Different letters within each row indicate means with significant treatment differences detected using Tukey-Kramer post hoc test, *P <* 0.05. One-sample *t*-test for comparing week 4 and week 8 mean change from baseline to the baseline of zero in each treatment group, with asterisks indicating a significant mean change from baseline (∗ *P <* 0.05; ∗∗ *P <* 0.001; ∗∗∗ *P <* 0.0001). Three-way ANOVA to determine treatment (*P*>0.0008), time (*P <* 0.001), sex (*P* > 0.20), and their interaction (*P* > 0.01) effects on the changes from baseline, with Tukey-Kramer post hoc test to determine statistical differences (*P <* 0.05). Abbreviations: ANOVA, Analysis of variance; 3D-AL, 3 dairy ad libitum; 3D-EN, 3 dairy energy neutral; LD-ER, low dairy energy restriction.

Protein intake was affected by week (*P =* 0.0018) and treatment-by-week (*P =* 0.032) interaction, but not by treatment (*P =* 0.059). Mean total protein intake was higher (*P =* 0.017) in 3D-EN (102.98 ± 4.77 g) than LD-ER (93.13 ± 3.86 g). Average intake in 3D-AL was 103.41 ± 7.58 g but was not statistically different from the other groups (*P =* 0.083). Females had 30.55 ± 2.69 g lower intake than males (*P =* 0.0056). The interaction between treatment and time is explained by the following. The change at week 8 (+18.29 ± 7.01 g) was different from baseline (*P =* 0.0012) and the change at week 4 (+6.44 ± 4.45 g; *P =* 0.041). In 3D-AL, intake increased by 10.29 ± 4.18 g at week 4 compared with week 0 (*P =* 0.036). In 3D-EN, there was a significant increase by 35.04 ± 8.18 g at week 8 from baseline (*P =* 0.012). There was also a significant difference in the change at week 8 between LD-ER, which decreased by 2.14 ± 9.42 g and 3D-EN (*P =* 0.017).

Fat intake was not impacted by treatment (*P =* 0.084) and week (*P =* 0.087) effects, but there were treatment-by-week interactions (*P =* 0.037). At week 8, there was a significant increase in fat intake by 31.14 ± 6.14 g from baseline (*P <* 0.001) in 3D-EN, but no significant changes from baseline were found in other treatment groups. This week 8 increase in 3D-EN was different from the decrease by 4.84 ± 10.60 g in LD-ER (*P =* 0.0079). The average daily fat intake in LD-ER (91.13 ± 5.84 g) was lower (*P <* 0.03) than 3D-EN (97.63 ± 4.55 g) but not different from 3D-AL (89.43 ± 4.80 g).

Calcium intake was significantly affected by treatment (*P =* 0.0009), week (*P =* 0.0013), and treatment-by-week (*P =* 0.014) effects. There was a mean increase by 506.59 ± 176.52 mg in 3D-AL, which was different from the 20.32 ± 57.91 mg decrease observed in LD-ER (*P =* 0.0013). The mean increase in 3D-EN by 284.96 ± 59.94 mg was intermediate. The mean increase in calcium intake at week 4 by 180.49 ± 41.39 mg was not significantly different from baseline (*P =* 0.056), but the increase by 236.66 ± 70.41 mg in 3D-EN (*P =* 0.0028) and by 371.74 ± 60.37 mg in 3D-AL (*P <* 0.0001) were significant and were different (*P =* 0.0006) from the change in LD-ER. A significant increase from baseline in calcium intake by 276.79 ± 120.46 mg was observed at week 8 (*P =* 0.001). This week 8 change was significantly different (*P =* 0.0002) between the LD-ER and 3D-AL groups, with a 47.67 ± 77.62 mg decrease in LD-ER and a 758.59 ± 417.01 mg increase in 3D-AL. Overall, daily calcium intake was significantly higher in 3D-EN (1100.10 ± 45.20 mg; *P =* 0.045) and 3D-AL (1120.50 ± 137.53; *P =* 0.0004) than LD-ER (760.28 ± 40.57 mg).

**FIGURE 4:**
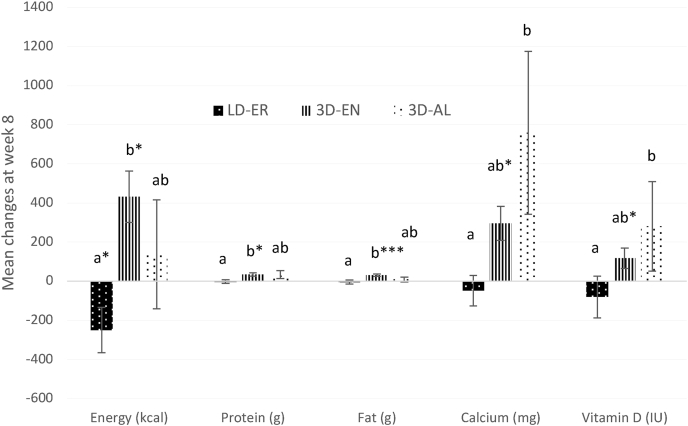
Changes in daily energy (kcal), protein (g), fat (g), calcium (mg), and vitamin D (IU) intake from baseline to week 8. Only outcomes with a significant treatment effect are illustrated; results for all other nutrients are presented in Table 8. Values are means ± SEM (*n =* 72). Treatment, time, and treatment-by-time effects were assessed by 3-way ANOVA with sex included as a factor. Tukey-Kramer post hoc test was used for pairwise comparisons as indicated by different letters (*P <* 0.05). Asterisks indicate significant within-group changes from baseline (∗ *P <* 0.05, ∗∗∗ *P <* 0.0001). Abbreviations: ANOVA, Analysis of variance; 3D-AL, 3 dairy ad libitum; 3D-EN, 3 dairy energy neutral; LD-ER, low dairy energy restriction.

No treatment group differences (*P* > 0.08), changes from baseline (*P* > 0.06), or treatment-by-week interaction effects (*P* > 0.20) were found for the changes from baseline in vitamin A, B2, B12, and D, as well as magnesium and potassium intakes. However, single-factor analyses presented in Table 8 showed significant increases in vitamin D intake in 3D-EN by 91.60 ± 32.51 IU at week 4 (*P =* 0.01) and by 117.10 ± 51.58 IU at week 8 (*P =* 0.034). The increase at week 8 in 3D-EN by 117.10 ± 51.58 IU was different from the decrease by 79.81 ± 106.92 IU in LD-ER (*P =* 0.0021). Magnesium intake increased by 66.04 ± 27.07 mg at week 4 (*P =* 0.023) whereas potassium intake increased by 643.71 ± 252.21 mg at week 8 (*P =* 0.019) in 3D-EN. There was a greater increase in potassium intake in 3D-EN by 561.73 ± 406.74 mg during week 4 than in LD-ER and 3D-AL, which had comparable intakes (*P =* 0.0025). Figure 4 shows the mean changes in daily energy and selected nutrient intakes (protein, fat, calcium, and vitamin D) from baseline to week 8 by treatment group.

### Dietary compliance and nutrition knowledge

The mean compliance with dairy consumption over the 12 wk was 79.9% ± 3.2%, with 58.5% ± 4.7% consumed within the correct 7–10 min time window (*n =* 49). The compliance was 78.8% ± 1.8% for milk and 70.3% ± 1.7% for cheese and yogurt. There were no significant differences in compliance between the dairy treatment groups (*P* > 0.60) or sexes (*P* > 0.50). Adherence to dairy servings remained consistent across the 12-wk intervention, with no significant effect of week (*P =* 0.67). In contrast to week 0 (rating of 3.36 ± 0.39 on a 10-point scale), participants at week 12 reported greater understanding of the CFG (6.83 ± 0.23; *P <* 0.0001) and how to apply it in their daily lives. The reported level of knowledge of the food guide was 7.54 ± 0.19 at week 12. Although only 32% of the participants were aware of the release of the new 2019 food guide at the start of the study, 92% believed it was essential to learn about it at the end. At week 12, there was a 77% compliance rate with the dietary recommendations provided (5.4 ± 0.12 days a week), and 94% were willing to continue the dietary recommendations they received beyond the study (*n =* 67; Table 9).

**TABLE 9:** Knowledge, attitudes, and practices of Canada’s Food Guide (CFG) dietary recommendations

Question	Unit of response	Week 0	Week 12
Do you think you understand the CFG and know how to use it in your daily life?	Out of 10	3.36 ± 0.39	6.83 ±0.23∗∗∗
Do you think you have enough knowledge of CFG to make/keep up changes to your eating habits in the future?	Out of 10	–	7.54 ± 0.19
Did you know that Health Canada released a new food guide in 2019?	Yes	32%	–
	No	68%	–
Do you feel it is essential to learn about CFG?	Yes	78%	92%
	No	22%	8%
Will you be continuing the eating plan or recommendation that were given to you?	Yes	–	94%
	No	–	6%
How often did you follow the dietary recommendation that were given?	Out of 7 days	–	5.40 ± 0.12

Data are presented as means ± SEM or as a percentage of total responses (*n* = 67). Asterisks indicate a significant difference (*P* < 0.05) from baseline determined through paired *t*-test (∗∗∗ *P* < 0.0001).

## Discussion

The results support our hypothesis that adding 3 servings of full-fat dairy combined with counseling to follow the CFG would not adversely affect the blood biomarkers of chronic disease but would increase intake of limiting nutrients and decrease intake of food and beverages associated with chronic diseases. They provide evidence that 3 servings of full-fat dairy can be accommodated in the diet of Canadians, within the context of the Canadian Food Guide. Three daily servings of full-fat dairy did not increase BMI, weight, body fat, HbA1c, BG, or lipids over 12 wk, when compared with an energy-restricted diet with low dairy consumption. Reductions in systolic blood pressure and HC, as well as higher limiting nutrient intakes, were found in the dairy consuming groups, whereas all participants made dietary changes in accordance with CFG over the 12 wk.

Adherence to the treatments and dietary guidance was shown by several lines of evidence. The overall adherence to dairy intake was high (79%), meeting our target of 3 servings in the high dairy groups and 1 serving in the low dairy group. Adherence to dietary counseling was indicated by increases and decreases in intake of foods and beverages as recommended by CFG and dietary guidance. The LD-ER group achieved an average decrease of 213 kcal rather than the intended 500 kcal. BW and BMI decreased in the LD-ER group when compared with the 3D-EN group.

In the 3D-EN group, energy neutrality was not achieved and was reflected in the small weight increase compared with the LD-ER group. HC was lower than baseline at week 12 in 3D-AL and BW, and BMI did not change, indicating that appetite regulation adjusted for the additions. The functionality of the dairy matrix with complex binding of fat, protein, lactose, calcium, and other nutrients may explain why participants did not gain weight despite a marked increase in full-fat dairy consumption [32]. Dairy protein, fats, and calcium have unique metabolic and physiological properties. In a comparison of the effects of individual macrocomponents of dairy with whole milk on metabolic hormone responses, the effects of the whole were proven to be more than simply a sum of its components [33]. As well, the extra fats and proteins provided by dairy may lead to a satiating effect, as those on the ad libitum diet did not have substantially higher energy intake than those on the energy-neutral diet despite their caloric freedom. Previous studies have also demonstrated the role of dairy in reducing hunger and food intake [[34], [35], [36]].

Body composition was not different among the treatment groups. Consistent with no effect on FFM was the absence of change over time in RMR or creatinine excretion [37,38]. Similarly, a crossover study involving a 6-month intervention of a high (≥4 servings/d) and low (≤1 serving/d) dairy diet in overweight and obese adults found an increase in weight during the high dairy phase consistent with an initial higher energy intake, but overall, no final group differences in BW, fat, BMI, WC, HC, body composition and RMR [39]. In contrast, a meta-analysis of randomized control trials showed that adding 2–4 daily servings of dairy to the diets of overweight/obese adults resulted in greater FM loss and 75% higher lean mass retention in comparison to low dairy control diets, possibly explained by higher protein, calcium, and medium-chain TGs intakes which have roles in regulating energy metabolism and satiety [9].

The lower SBP in LD-ER and 3D-AL groups at the end of the study is also consistent with other reports, suggesting that further exploration of the effect of dairy fat and full-fat dairy on blood pressure regulation is merited [40,41]. Furthermore, although the Dietary Approaches to Stop Hypertension diet recommends 2 servings of low-fat dairy each day, a 12-wk study of adults with metabolic syndrome found a SBP reduction in the group with 3 daily servings of low-fat dairy [12,42].

Dietary counseling to encourage the participants to follow CFG was effective. Over the course of 12 wk, the participants received a total of 120 min of nutrition counseling. It led to a doubling in the understanding and application of the CFG by the end of the study, whereby 94% of participants expressed willingness to continue the recommended eating pattern in the future. The dietary shifts aligned with the CFG, including an increase in whole grains and a decrease in animal proteins, ruminant meats, fats, processed and confectionery foods, unsweetened beverages, and alcohol. The CFG recommends consuming half of grain foods as whole grains, reducing animal-based foods, limiting fat intake to 2–3 tbs of unsaturated fats, eliminating processed and confectionery foods, and selecting water as the drink of choice [19,31]. However, participants did not increase their fruit and vegetable intake, nor their intake of plant proteins. Overall, participants consumed fewer than 4 servings of fruits and vegetables and plant protein foods instead of the recommended 7–10 servings/d, comparable to the national average of 4.5 servings/d [31,43]. Males consumed more servings of fruits and vegetables and plant proteins in this study than females, which may be associated with their overall higher energy intake rather than choice. In addition, there were no improvements in food skills [19]. Although participants reported eating home-cooked meals an average of 83% of the time, they need encouragement to improve cooking methods and to read food labels.

During the first 8 wk of the study, increases in the intakes of energy, protein, fat, calcium, vitamin D, potassium, and magnesium were seen among the dairy consuming participants but not in the LD-EN group. However, differences in protein intake were not reflected in urea excretion, which was similar for all groups. This can be explained by the use of single spot-check samples of urine collected from fasted participants attending the research center. Urinary nitrogen output reflects ≤80% of dietary protein intake, based on 24-h urine nitrogen output over several days [44]. Fat accounted for 42% of daily calories in the low dairy group and 38% in the high dairy group, above the recommended 20%–35% [45].

The dairy groups increased their average intake of calcium above the recommended target of 1000 mg, whereas the LD-ER each day averaged only 760 mg/d [46]. Calcium consumption was 1120 mg in 3D-AL and averaged 1110 mg/d in the high dairy consuming groups. There were no significant changes in vitamin A, B2, and B12 intakes over the timeframe of the study. Despite the mandatory fortification of milk with vitamin D in Canada, intakes were below the Recommended Dietary Allowance of 600 IU. However, as of 2022, the fortification requirement of vitamin D in milk has been doubled to 2 μg per 100 mL, and voluntary fortification of yogurt and kefir has also been permitted. When applied by 2025, this will increase the effectiveness of dairy to meet vitamin D requirements [47]. Magnesium and potassium intakes were below the daily dietary allowance before the study, but intake increased to meet the requirement for females in the 3D-EN and 3D-AL groups, respectively [48].

The strength of the results of this study for application to Canadian dietary recommendations resides with the novel approach of adding 3 servings of dairy to the diets of obese and overweight participants who made adjustments in their diets that were consistent with CFG. The results align with the conclusion of an expert group that there is no evidence to support the avoidance of high-fat dairy in diets [18].

The weakness of this study was presented by the COVID-19 limitations on the recruitment of the targeted sample size of 50 participants per group, the carryout of the study length of 24 wk, and the termination of funding due to government timelines. Nevertheless, the achieved sample size provides sufficient evidence to justify a repeat study of a larger sample size and duration. It was sufficiently powered to detect changes in fasting BG, lipids, and HbA1c over 12 wk [49,50]. However, the short duration of the study may have masked longer-term changes in these measures and in BMI and body composition as well.

Another limitation of the present study is that circulating fatty acid profiles were not assessed. Specific bioactive fatty acids found in dairy fat, including conjugated linoleic acid, vaccenic acid, and long-chain n-3 (omega-3) fatty acids such as DHA, have been shown in controlled interventions to influence lipid metabolism beneficially [51,52]. Future studies that include detailed fatty acid profiling may provide mechanistic insights into the cardiometabolic effects of dairy consumption.

In summary, this study examined the long-term metabolic and nutritional impacts of regular consumption of full-fat dairy accompanied by dietary counseling to follow Canada’s Food Guide. We found that consuming 3 daily servings of full-fat dairy did not lead to increases in weight, body fat, HbA1c, BG or lipids when compared with an energy-restricted diet with low dairy consumption. Improvements in systolic blood pressure, HC, BMI, and potentially limiting nutrient intakes were found in the dairy consuming groups, whereas all participants made dietary changes in accordance with the food guide over the 12 wk.

In conclusion, 3 daily servings of full-fat dairy can be accommodated by Canada’s Food Guide 2019 and play a supportive role in meeting dietary recommendations and requirements.

## Author contributions

The authors’ responsibilities were as follows – GHA, HF, BL: designed research; MS, LC, MA: conducted research; SV, PK, FS: provided support and oversight for data collection; CZCZ, MS, SV: analyzed data; GHA, CZCZ, SV, MS: wrote article; SS: edited article; GHA: primary responsibility for final content; and all authors: read and approved the final manuscript.

## Data availability

Data described in the manuscript, code book, and analytic code will be made available on request pending (e.g., application and approval, payment, other).

## Funding

This research was supported by Dairy Research Cluster 3 (Dairy Farmers of Canada and Agriculture and Agri-Food Canada) under the Canadian Agricultural Partnership AgriScience Program, and the Mitacs Accelerate program. The supporting sources were not involved and presented no restrictions in the publication of this research.

## Conflict of interest

The authors report no conflicts of interest.
